# HDAC4 Knockdown Alleviates Denervation-Induced Muscle Atrophy by Inhibiting Myogenin-Dependent Atrogene Activation

**DOI:** 10.3389/fncel.2021.663384

**Published:** 2021-06-30

**Authors:** Wenjing Ma, Yong Cai, Yuntian Shen, Xin Chen, Lilei Zhang, Yanan Ji, Zehao Chen, Jianwei Zhu, Xiaoming Yang, Hualin Sun

**Affiliations:** ^1^Key Laboratory of Neuroregeneration of Jiangsu and Ministry of Education, NMPA Key Laboratory for Research and Evaluation of Tissue Engineering Technology Products, Jiangsu Clinical Medicine Center of Tissue Engineering and Nerve Injury Repair, Co-Innovation Center of Neuroregeneration, Nantong University, Nantong, China; ^2^Department of Neurology, People’s Hospital of Binhai County, Yancheng, China; ^3^Department of Neurology, Affiliated Hospital of Nantong University, Nantong, China; ^4^Department of Orthopedics, Affiliated Hospital of Nantong University, Nantong, China

**Keywords:** muscle atrophy, HDAC4, myogenin, CDKN1A, SIK1

## Abstract

Denervation can activate the catabolic pathway in skeletal muscle and lead to progressive skeletal muscle atrophy. At present, there is no effective treatment for muscle atrophy. Histone deacetylase 4 (HDAC4) has recently been found to be closely related to muscle atrophy, but the underlying mechanism of HDAC4 in denervation-induced muscle atrophy have not been described clearly yet. In this study, we found that the expression of HDAC4 increased significantly in denervated skeletal muscle. HDAC4 inhibition can effectively diminish denervation-induced muscle atrophy, reduce the expression of muscle specific E3 ubiquitin ligase (MuRF1 and MAFbx) and autophagy related proteins (Atg7, LC3B, PINK1 and BNIP3), inhibit the transformation of type I fibers to type II fibers, and enhance the expression of SIRT1 and PGC-1 α. Transcriptome sequencing and bioinformatics analysis was performed and suggested that HDAC4 may be involved in denervation-induced muscle atrophy by regulating the response to denervation involved in the regulation of muscle adaptation, cell division, cell cycle, apoptotic process, skeletal muscle atrophy, and cell differentiation. STRING analysis showed that HDAC4 may be involved in the process of muscle atrophy by directly regulating myogenin (MYOG), cell cycle inhibitor p21 (CDKN1A) and salt induced kinase 1 (SIK1). MYOG was significantly increased in denervated skeletal muscle, and MYOG inhibition could significantly alleviate denervation-induced muscle atrophy, accompanied by the decreased MuRF1 and MAFbx. MYOG overexpression could reduce the protective effect of HDAC4 inhibition on denervation-induced muscle atrophy, as evidenced by the decreased muscle mass and cross-sectional area of muscle fibers, and the increased mitophagy. Taken together, HDAC4 inhibition can alleviate denervation-induced muscle atrophy by reducing MYOG expression, and HDAC4 is also directly related to CDKN1A and SIK1 in skeletal muscle, which suggests that HDAC4 inhibitors may be a potential drug for the treatment of neurogenic muscle atrophy. These results not only enrich the molecular regulation mechanism of denervation-induced muscle atrophy, but also provide the experimental basis for HDAC4-MYOG axis as a new target for the prevention and treatment of muscular atrophy.

## Introduction

Peripheral nerve injury is bound to cause the loss of target muscle autonomic contraction function and insufficient blood supply, and then trigger a series of pathophysiological reactions in skeletal muscle, including oxidative stress, inflammation, mitochondrial autophagy and enhanced catabolism, etc. (Čebašek, 2016; Ma et al., 2018, 2019; Huang et al., 2019; Scalabrin et al., 2019; Shen Y. et al., 2019; Yu et al., 2020). Although the molecular mechanism of skeletal muscle atrophy induced by peripheral nerve injury is not yet understood, the current widely accepted theory is that loss of innervation in skeletal muscle disrupts homeostasis of protein synthesis and proteolysis. The rate of proteolysis is higher than the rate of protein synthesis, resulting in the proteolysis of a large number of structural proteins and the loss of muscle fiber (Bodine et al., 2001; Yuan et al., 2015; Machado et al., 2016; Lang et al., 2017). As the duration of skeletal muscle denervation lengthens, the denervated skeletal muscle fibers will synthesize a large amount of collagen, accumulating between muscle fibers, hindering the transmission of cell signals between muscle fibers. This in turn exacerbates skeletal muscle atrophy, causing irreversible muscle atrophy and fibrosis, with a high risk of disability (Contreras et al., 2016; Liu et al., 2016; Feng et al., 2019). Due to the very slow rate of peripheral nerve regeneration (1 mm/d), irreversible atrophy often occurs before skeletal muscle regaining nerve reinnervation. In conclusion, intervention in the early stage of denervated skeletal muscle may achieve a satisfactory effect of decreasing muscle atrophy. Therefore, we need to find the triggers of skeletal muscle atrophy in the early stage of denervation and increase/decrease the triggers to alleviate the progression of skeletal muscle atrophy, gaining time for the regeneration of the nerve. This is the focus of current research.

Histone deacetylase 4 (HDAC4) is a key enzyme that regulates chromosome structure and gene expression. In the nucleus, HDAC4 removes acetyl groups from histones, causing them to be deacetylated. This in turn causes chromatin to become dense and curly, inhibiting gene transcription, which controls the important process of cell life activities (Huynh et al., 2017). HDAC4 can promote the proliferation of muscle satellite cells by regulating transcription factors in skeletal muscle (Choi et al., 2014). On the other hand, HDAC4 is believed to be an inhibitor of cell differentiation, and inhibition of HDAC4 can promote differentiation of muscle satellite cells (Bharathy and Taneja, 2012). Different phosphorylation and localization of HDAC4 play an important role in maintaining the transcription of genes related to different muscle fiber types. HDAC4 is predominantly localized to the nuclei in fast-twitch fibers, while the cytoplasmic localization is associated with HDAC4 hyper-phosphorylation in slow-twitch fibers (Cohen T. J. et al., 2015). HDAC4 is highly expressed in many neurogenic myopathies (Cohen et al., 2009; Bricceno et al., 2012; Pigna et al., 2018, 2019; Federspiel et al., 2019). HDAC4 inhibitors can significantly improve the motor function of mouse models of amyotrophic lateral sclerosis (ALS; Pigna et al., 2019), but complete deletion of HDAC4 will aggravate the myopathy of ALS (Cohen et al., 2009), which may be related to the role of HDAC4 in maintaining the integrity of normal neuromuscular junctions. Regulation of HDAC4 expression by miRNA-206 can effectively improve the ability of motor neurons to control muscle in SMA (spinal muscular atrophy) patients, and maintain the shape and number of the motor endplate in skeletal muscle (Bricceno et al., 2012). Meanwhile, miRNA-206 can also diminish denervation-induced muscle atrophy by regulating HDAC4 (Huang Q. K. et al., 2016). In a mouse model of denervated muscle atrophy, combined knockout of HDAC4 and HDAC5 could significantly reduce the loss of muscle mass, while knockout of HDAC4 alone also partially decreased denervation-induced muscle atrophy, which suggested that HDAC4 might play an important role in denervation-induced muscle atrophy (Moresi et al., 2010). HDAC4 has been shown to increase denervation-induced muscle atrophy through GADD45α (Bongers et al., 2013). Meanwhile, HDAC4/GADD45α axis also plays an important role in the prevention of muscle atrophy induced by hind limb unloading in elderly rats (Yoshihara et al., 2019). Recent studies have shown that HDAC4 and HDAC5 are involved in muscle atrophy induced by hind limb unloading through the regulation of myogenin (MYOG), ubiquitin, and calpain-1 (Mochalova et al., 2020). In conclusion, the role of HDAC4 in skeletal muscle cells is complex, and there are many regulatory molecular targets.

In order to further clarify the role of HDAC4 and the downstream targets of HDAC4 in denervation-induced muscle atrophy, the HDAC4 knockdown model was constructed to investigate the role of HDAC4 in denervation-induced muscle atrophy by muscle targeted injection with lentivirus expressing HDAC4 shRNA, and the downstream targets of HDAC4 in denervation-induced muscle atrophy were explored by transcriptome sequencing and bioinformatics analysis. Through the study of the potential mechanism of HDAC4 in the process of muscle atrophy, we can provide an experimental basis for the discovery of new targets for the prevention and treatment of denervation-induced muscle atrophy.

## Materials and Methods

### Animal Experiment

Animal experiments were conducted in accordance with the Animal Care Guidelines of Nantong University and approved by Jiangsu Provincial Laboratory Animal Management Committee (No. 20180305-004; date: January 01, 2019 to December 12, 2022). Eight-week-old male ICR mice, weighing 25 ± 2 g, were provided by Laboratory Animal Center, Nantong University. The animals were kept at 22°C, 12 h in the light/dark circulation environment, and their movement and feeding were not restricted. ICR mice were randomly divided into six mice in each group. Left tibialis anterior muscle was exposed in anaesthetized mice, and anesthesia methods refer to our previous study (Shen Y. et al., 2019). In order to detect the expression of HDAC4 in skeletal muscle at different time points after denervation, the tibialis anterior muscles were removed at 3 days, 7 days, 14 days and 28 days after denervation. To investigate the effect and mechanism of HDAC4 on denervated skeletal muscle, Lentivirus (25 μl, 1 × 10^8^ TU) expressing HDAC4-shRNA (H-i+Den group), myogenin-shRNA (G-i+Den group), HDAC4-shRNA+myogenin overexpression (H-i/G-oe+Den group) was injected into the tibialis anterior muscle with multipoint injection. Lentivirus vector for shRNA: GV298, component sequence: U6-MCS-Ubiquitin-Cherry-IRES-puromycin; Lentivirus vector for overexpression: CV186, component sequence: Ubi-MCS-3FLAG-SV40-Cherry-IRES-puromycin. Sham group and denervated group were injected with the same amount of empty vector virus as control. Three days later, a 10-mm defect was made in the left sciatic nerves of the experimental group, and the proximal sciatic nerve was folded and sutured subcutaneously to create the sciatic nerve defect model, which was used as the denervated model in this study. In the sham group, only the sciatic nerve was exposed without dissection. After 14 days, the bilateral tibialis anterior muscles of mice in each group were taken, weighed and the wet weight ratio (the ratio of the muscle mass on the operative side to that on the contralateral side) was calculated. The weighed muscles were frozen in a −80°C freezer for further study, or fixed with formaldehyde/glutaraldehyde for further study.

### Immunofluorescence Staining

The tibialis anterior muscle fixed with paraformaldehyde was prepared into frozen sections with a thickness of 8 μm (Huang et al., 2019). Sections were placed on slides, and sealant solution was used for 60 min, then the sections were incubated with anti-laminin (ab11575, 1:200, Abcam)/anti-fast Myosin Heavy chain antibodies (ab91506, 1:200, Abcam) overnight at 4°C. The slides were rinsed with PBS and incubated with goat polyclonal secondary antibody to rabbit IgG-H&L (ab150077, 1:200, Abcam) at room temperature for 2 h. The images were taken by fluorescence microscopy (ZEISS). Five fields were randomly selected for laminin staining, and the cross-sectional area of muscle fibers was counted. The results of fast myosin skeletal heavy chain staining were analyzed to evaluate the proportion of fast muscle fibers in the muscle.

### Transmission Electron Microscopy (TEM) Analysis

The detailed procedures of Transmission Electron Microscopy (TEM) for muscle were previously reported (Sun et al., 2016). Briefly, immediately after the mice were sacrificed, the TA muscles were dissected, and rinsed in phosphate buffer solution. Sections of muscle 1 mm^3^ in volume were fixed in pre-cooled glutaraldehyde (2.5%) followed by postfixation in 1% osmium tetroxide for the assays of electromicroscopy. The fixed muscle was made into ultrathin sections, and the images were observed by TEM (HT7700, Tokyo, Japan), and the ultrastructural changes of muscle fibers and mitochondria were analyzed.

### Western Blot

Total protein was extracted from frozen muscle tissue or C2C12 myotubes with radio immunoprecipitation assay (RIPA) buffer (P0013C, Beyotime, Haimen, China), and the concentration of total protein was detected by BCA protein concentration determination kit (P0010, Beyotime, Haimen, China). Then the total proteins were separated by SDS-PAGE, and the proteins on the gel were transferred to the PVDF membrane by electrotransfer method. The PVDF membrane was rinsed with TBST and incubated overnight with corresponding antibodies at 4°C. The primary antibodies included mouse anti-myosin heavy chain (MHC) polyclonal antibody (R&D Systems, Minneapolis, MN), rabbit antibodies against PGC-1α (Thermo Fisher Scientific; PA5-38022, dilutions, 1:2,000) and MuRF1 (Thermo Fisher Scientific; PA5-96226, dilutions, 1:2,000), rabbit antibodies against MAFbx (Fbx32; ab168372; dilutions, 1:1,000), LC3B (ab48394; dilutions, 1:1,000), PINK1(ab23707; dilutions, 1:1,000), BNIP3 (ab10433; dilutions, 1:1,000), ATG7 (ab133528; dilutions, 1:1,000), Sirt1 (ab110304; dilutions, 1:1,000), HDAC4 (ab12172; dilutions, 1:1,000), MYOG (ab124800, dilutions, 1:2,000), and beta-tubulin (ab6046; dilutions, 1:1,000; Abcam, Cambridge, UK). The next day, the PVDF membrane was incubated at room temperature with the corresponding secondary antibody (HRP-conjugated Goat anti-Mouse IgG, LS-C60710, 1:5,000; HRP-conjugated Goat anti-Rabbit IgG, LS-C60884, 1:5,000) coupled with HRP for 2 h. A Chemiluminescence imaging system is used to detect gray stripe, and Image J software was used to analyze the grayscale of the strips. Beta-tubulin was used as the internal reference.

### Transcriptome Sequencing and Bioinformatics Analysis

Total RNA was extracted from tibialis anterior muscle with mirVana™ miRNA ISOlation Kit (Ambion-1561) and its quality was detected. The ribosomal RNA was digested using the TruSeq Stranded Total RNA with Ribo-Zero Gold kit, and an interrupting reagent was added to break the RNA into short fragments. The interrupted RNA was used as a template to synthesize a strand of cDNA with First-Strand Synthesis Act D Mix and SuperScript II Reverse Transcriptase to construct an RNA library. Agilent 2100 BioAnalyzer was used for quality control, and Illumina sequencer was used for sequencing. DESeq software was used to normalize the counts of mRNA in each sample, and the different multiple was calculated. NB (negative binomial distribution test) was used to test the difference significance of reads, and the different genes were screened according to log2 (fold change) <−1 or >1 and *P* value <0.001. Furthermore, the genes up-regulated or down-regulated after denervation (compared with the Sham group) were intersected with the genes down-regulated or up-regulated after HDAC4 interference (compared with the Den group) to obtain the differentially expressed genes. GO (Gene ontology) and STRING analysis (functional protein association networks) were performed for different genes. The gene expression dataset is available on ArrayExpress and the accession number is E-MTAB-10072.

### Differentiation and Virus Transfection of C2C12 Cells

The C2C12 cell lines were obtained from the American Type Culture Collection, cultured at 37°C and 5% CO_2_. The C2C12 cell lines were cultured in proliferation medium (10% FBS with 100 units of penicillin and streptomycin antibiotics). When the cell density reached about 80%, C2C12 cells were induced to differentiate into myotubes by replacing the proliferation medium with the differentiation medium (2% horse serum with 100 units of penicillin and streptomycin antibiotics), and transfected with proliferation medium on the third day of differentiation. C2C12 myotubes were exposed to viral vectors (1.5 × 10^6^ TU/μl, myogenin overexpression) for 24 h. The control group was transfected with an empty vector virus, and the fresh medium was replaced 24 h later. Microscopic observation was performed 3–4 days after the viral vector was transfected into C2C12 cells.

### C2C12 Myotube Diameter

The C2C12 myotubes were fixed with 4% paraformaldehyde for 20 min, and then the C2C12 myotubes were incubated with Immunol Staining Blocking Buffer (P0102, Beyotime, Haimen, China) for 60 min. The C2C12 myotubes were rinsed with PBS and incubated overnight with Myosin Heavy Chain Antibody (MAB4470, 1:200, R&D) at 4°C. The rinsed C2C12 myotubes were incubated at room temperature with goat polyclonal secondary antibody to mouse IgG-H&L (ab150113, 1:600, Abcam) for 2 h. After rinsing and sealing, the C2C12 myotubes were observed and photographed under a fluorescence microscope (ZEISS), and the diameter of the myotubes was measured by Image J software (NIH).

### Statistical Analysis

All data in this study were expressed as mean ± standard deviation. All data were analyzed by one-way analysis of variance, and Tukey’s multiple comparisons test was used to detect differences between groups. All statistical analyses in the current study were performed using GraphPad Prism software (version 7.0; San Diego, CA, USA). *P* < 0.05 was considered statistically significant.

## Results

### HDAC4 Inhibition Effectively Diminishes Denervation-Induced Muscle Atrophy

**Figure 1:**
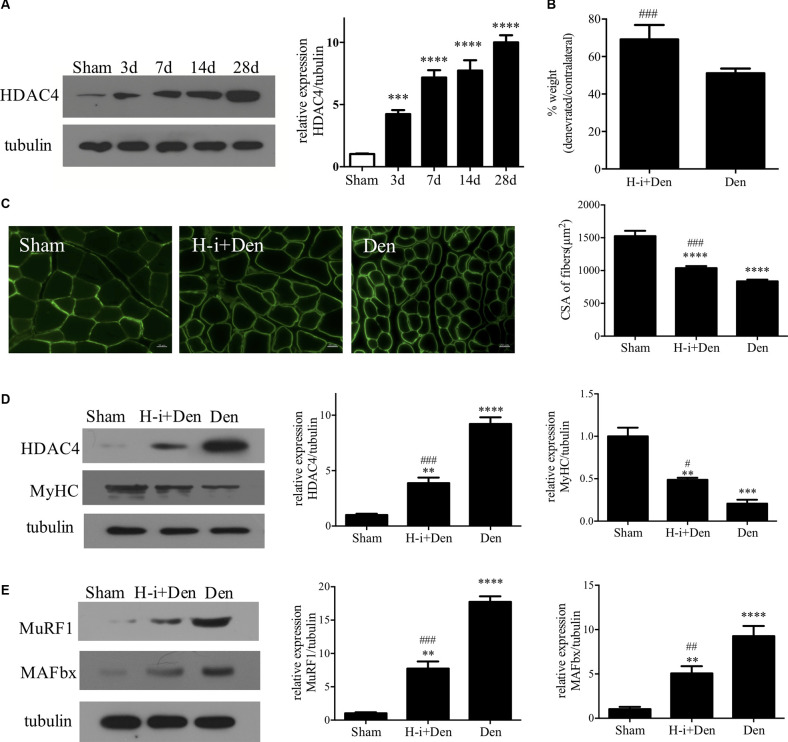
HDAC4 inhibition diminished denervation-induced muscle atrophy. **(A)** The expression of HDAC4 in tibialis anterior muscle at different time points after denervation was detected by Western blot. **(B)** Wet weight ratio of muscle. Sciatic nerve transection model was prepared after injection of HDAC4-shRNA lentivirus (H-i+Den) or empty vector virus (Den) into the tibialis anterior muscle of mice. The empty vector virus was injected into muscles from the sham group (Sham). Muscle wet weight ratio was measured after 14 days. **(C)** The muscle fiber cross section area (CSA) was analyzed by laminin staining. **(D)** The expression levels of HDAC4 and MyHC were detected by Western blot. **(E)** The expression levels of muscle specific E3 ubiquitin ligase (MuRF1 and MAFbx) were detected. **P < 0.01, ***P < 0.001, ****P < 0.0001 vs. sham group. ^#^*P* < 0.05, ^##^*P* < 0.01, ^###^*P* < 0.001 vs. denervated group.

HDAC4 expression was detected in skeletal muscle at different time points after denervation, and the results showed that HDAC4 expression continued to increase in skeletal muscle after denervation (Figure 1A). After HDAC4 shRNA interference, the wet weight ratio and muscle fiber cross-sectional area of mice were significantly higher than those of the denervated group (Figures 1B,C). Myosin heavy chain (MyHC) is an important functional component in muscle, which plays an important role in maintaining the normal function of muscle cells. Our results showed that MyHC expression was significantly decreased in skeletal muscle after denervation, and HDAC4 inhibition significantly reversed the denervation-induced decrease in MyHC expression (Figure 1D). These results suggest that HDAC4 inhibition reduces the denervation-induced hydrolysis of MyHC. To further evaluate the protective effect of HDAC4 inhibition on muscle, we detected the expression of the ubiquitinated proteasome hydrolysis system in the tibialis anterior muscle. MuRF1 and MAFbx are specific E3 ubiquitin ligases in muscle, and their high expression leads to massive hydrolysis of structural and functional proteins in muscle (Sanchez et al., 2014; Khalil, 2018; Nowak et al., 2019). In this study, we found that the expressions of MuRF1 and MAFbx were significantly down-regulated in the muscle of mice with HDAC4 inhibition compared with the DEN group (Figure 1E), which suggested that HDAC4 inhibition may decrease the activity of the ubiquitin proteasome pathway. These results indicate that HDAC4 expression is increased in skeletal muscle after denervation, and HDAC4 inhibition can effectively diminish denervation-induced skeletal muscle atrophy.

### HDAC4 Inhibition Alleviates Mitophagy and Muscle Fiber Type Transition From Slow-Fast Switch

**Figure 2:**
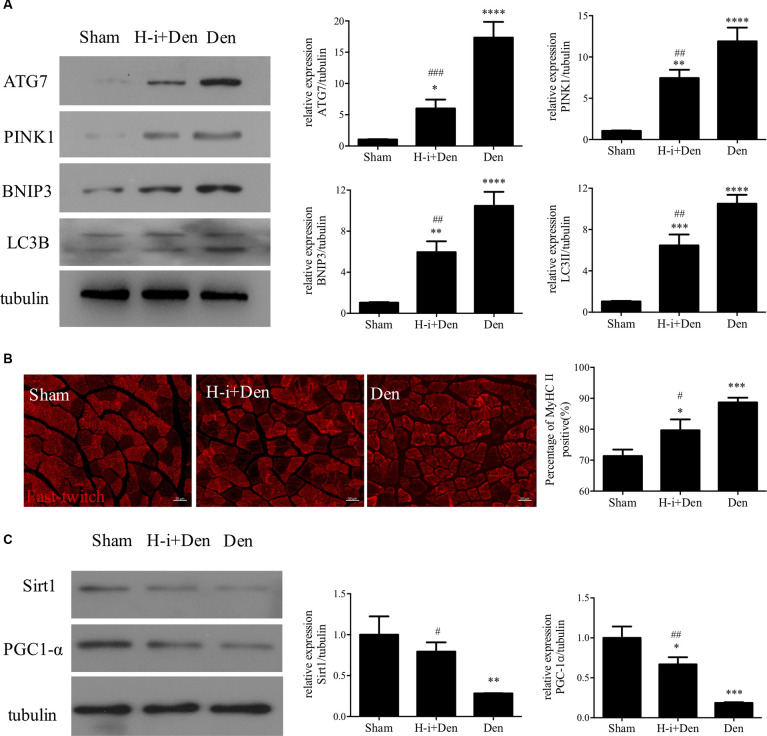
HDAC4 inhibition reduced the expression of autophagy-related proteins and retarded muscle fiber type transformation in denervated skeletal muscle. **(A)** The expression of autophagy-related proteins in tibialis anterior muscle was detected by Western blot. The sciatic nerve transection model was prepared after injection of HDAC4-shRNA lentivirus (H-i+Den) or empty vector virus (Den) into the tibialis anterior muscle of mice. The empty vector virus was injected into muscles from the sham group (Sham). After 14 days, the expression of autophagy-related proteins in tibialis anterior muscle was detected by Western blot. **(B)** MyHC II fiber staining was used to analyze the fast muscle fibers in the tibialis anterior muscle after denervation and the positive rate of MyHC II fiber was calculated. **(C)** The expression changes of mitochondrial generation associated proteins (SIRT1 and PGC-1α) in tibialis anterior muscle were detected by Western Blot after denervation. *P < 0.05, **P < 0.01, ***P < 0.001, ****P < 0.0001 vs. sham group. ^#^*P* < 0.05, ^##^*P* < 0.01, ^###^*P* < 0.001 vs. denervated group.

Autophagy plays an important role in muscle homeostasis (Sandri, 2013). In the case of acute muscle injury, the autophagy-lysosome system can effectively remove the damaged components and harmful substances (such as ROS) and lay the foundation for muscle recovery (Di Rienzo et al., 2019). However, with the progression of denervation, the autophagy-lysosome system inevitably causes excessive loss of cellular components, which is not conducive to the subsequent recovery of skeletal muscle (Sandri, 2010). Western Blot results showed that the expression of autophagy-related proteins (Atg7, LC3B, PINK1 and BNIP3) was significantly increased in the denervated skeletal muscle, and HDAC4 inhibition could significantly suppress the high expression of Atg7, LC3B, PINK1 and BNIP3 in the denervated skeletal muscle (Figure 2A). These results indicate that HDAC4 inhibition can reduce mitophagy.

Abnormal mitochondrial function often affects oxidative metabolism and causes the transformation of slow muscle fibers to fast muscle fibers. Our results showed that the proportion of fast muscle fibers in the tibialis anterior muscle was significantly increased after denervation, while HDAC4 inhibition could suppress the increased proportion of fast muscle fibers in the denervated tibialis anterior muscle (Figure 2B). SIRT1 plays an important role in muscle remodeling, and SIRT1 can also increase the activity of PGC-1α to promote mitochondrial biogenesis (Shen S. et al., 2019). Activation of the SIRT1/PGC-1α pathway can promote the expression of the slow oxidative myogenic program, that is, the SIRT1/PGC-1α pathway can drive muscle fiber type conversion and directly increase the physiological function of skeletal muscle (Huang C. C. et al., 2016). Western Blot results showed that the expression of SIRT1 and PGC-1α in denervated skeletal muscle was significantly decreased, and HDAC4 inhibition could significantly activate the expression of SIRT1 and PGC-1α in denervated skeletal muscle (Figure 2C), which suggest that HDAC4 inhibition can improve mitochondrial function and inhibit slow muscle fibers to fast muscle fibers. In conclusion, HDAC4 inhibition can effectively reduce mitochondrial autophagy and decrease fiber type transformation after denervation.

### Effect of HDAC4 Inhibition on Transcriptome of Denervated Skeletal Muscle

**Figure 3:**
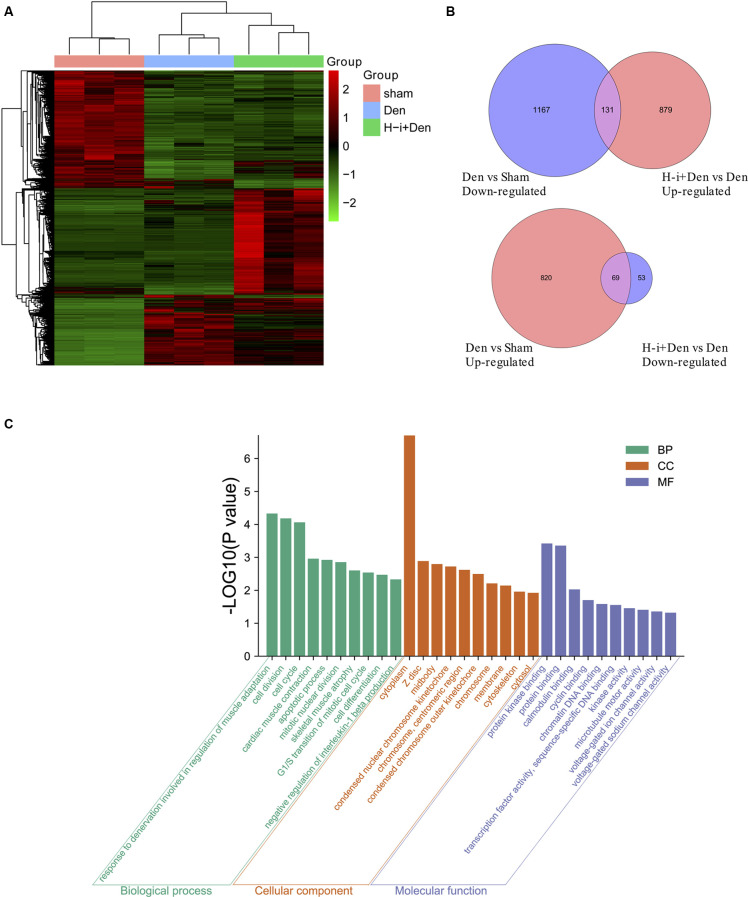
Effects of HDAC4 inhibition on the transcriptome of atrophied muscles induced by denervation. **(A)** Heat map of gene expression of tibialis anterior muscle in H-i+Den group (H-i+Den), den group (Den) and sham group (Sham) at 7 days after the operation. Clustering of all the differentially expressed genes (*P* < 0.001; A Fold change 2 or higher). The standardized expression level, from green to red, represents expression level from low to high, *n* = 3. **(B)** Venn diagrams of down-regulated genes after denervation and up-regulated genes after interference with HDAC4; Venn diagram of up-regulated genes after denervation and down-regulated genes after interference with HDAC4. **(C)** GO (Gene ontology) analysis was performed on further screened differentially expressed genes.

**Figure 4:**
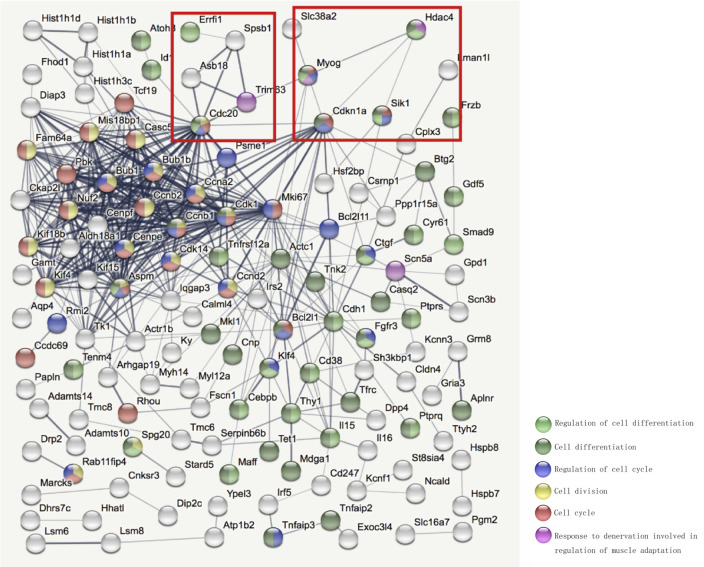
STRING (https://string-db.org/) was used to further analyze the interaction network among the selected differentially expressed genes. Bottom right corner of the image: Different colored balls represent different biological processes.

To investigate the molecular mechanism by which HDAC4 inhibition decreased denervation-induced muscle atrophy, transcriptome sequencing was performed on denervated muscle, and the expression of 2187 genes was significantly altered after denervation for 7 days. Compared with the denervation group, the expression of 1,132 genes significantly changed after HDAC4 interference (Figure 3A). The genes up-regulated or down-regulated after denervation were intersected with those up-regulated or up-regulated after HDAC4 interference respectively, and a total of 200 genes were obtained (Figure 3B, Supplementary Table 1). GO analysis of these 200 genes showed that the biological processes involved in these genes mainly involved cell division, cell cycle, cardiac muscle contraction, apoptotic process, skeletal muscle atrophy, and response to denervation involved in the regulation of muscle adaptation (Figure 3C). STRING analysis showed that HDAC4 was interrelated with MYOG, CDKN1A and SIK1 (Figure 4), and MYOG, CDKN1A and SIK1 were all involved in the regulation of the cell cycle and cell differentiation. The most interesting one is MYOG, which is not only related to muscle development, but also involved in response to denervation in the regulation of muscle adaptation. MYOG is associated with TRIM63 (MuRF-1), a muscle specific E3 ubiquitin ligase. ASB18 (Ankyrin repeat and SOCS box protein 18) and Spsb1 (SPRY domain-containing SOCS box protein 1) are directly related to TRIM63, and both ASB18 and Spsb1 are related to protein ubiquitination. These results suggest that MYOG regulates the expression of genes involved in ubiquitination and protein degradation. Our results showed that the expression of MYOG was significantly increased in skeletal muscle after denervation, and HDAC4 inhibition could significantly reverse the high expression of MYOG in skeletal muscle after denervation (Supplementary Figure 1).

### HDAC4 Inhibition Alleviates Denervation-Induced Muscle Atrophy by Down-Regulating MYOG

In order to explore the role of MYOG in the process of muscle atrophy, we first constructed MYOG lentiviral overexpression vector. In C2C12 myotubes, the diameters of MYOG overexpressed myotubes were significantly reduced compared with the control group (Supplementary Figures 2A,B). Overexpression of MYOG can significantly reduce MyHC expression and promote the expression of MuRF1 and MAFbx (Supplementary Figure 2C). These results suggest that MYOG, which is highly expressed in skeletal muscle after denervation, may be involved in the activation of the ubiquitin-proteasome system, thereby promoting muscular atrophy.

**Figure 5:**
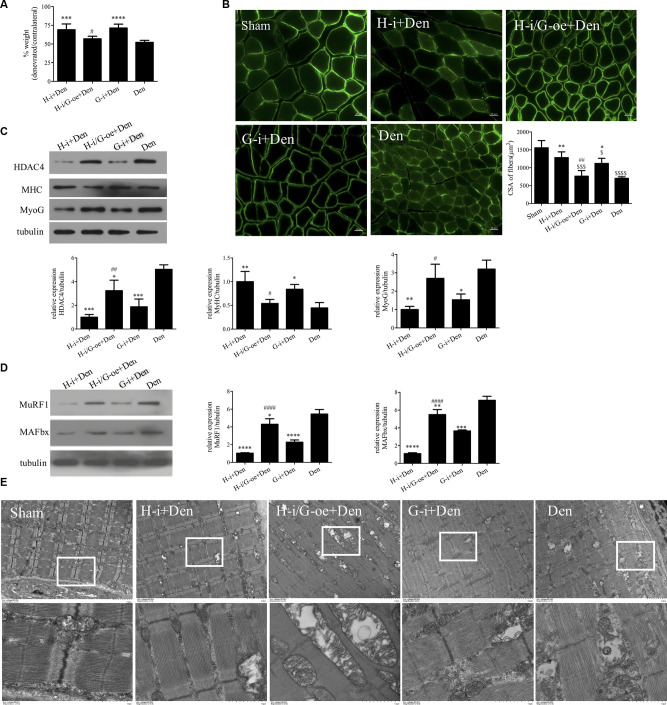
HDAC4 inhibition alleviated denervation-induced muscle atrophy by downregulating myogenin (MYOG). **(A)** The sciatic nerve transection model was prepared after injection of HDAC4-shRNA lentivirus (H-i+Den), HDAC4-shRNA+MYOG overexpression lentivirus (H-i/G-oe+Den), MYOG-shRNA lentivirus (G-i+Den) or empty vector virus (Den) into the tibialis anterior muscle of mice 3 days, and muscle weight ratio was measured after denervation for 14 days. **(B)** Laminin staining was used to analyze the cross-sectional area of muscle fibers. **(C,D)** Protein levels of HDAC4, MYOG, MyHC, MAFbx and MuRF1 in muscle were detected by Western blot. **(E)** Observation of mitochondrial cavitation degeneration and mitophagy in muscle fibers by Transmission Electron Microscopy (TEM). *P < 0.05, **P < 0.01, ***P < 0.001, ****P < 0.0001 vs. denervated group. ^#^*P* < 0.05, ^##^*P* < 0.01, ^####^*P* < 0.0001 vs. HDAC4-shRNA lentivirus group. ^$^*P* < 0.05, ^$$$^*P* < 0.001, ^$$$$^*P* < 0.0001 vs. sham group.

In the above study, we found that MYOG is elevated and regulated by HDAC4 after denervation, and high levels of MYOG can cause C2C12 myotubes atrophy. In order to further clarify the relationship between HDAC4 and MYOG and its role in denervation-induced muscle atrophy, we compared the denervation group (Den) and the denervation group interfering with MYOG (G-i+Den), the denervation group interfering with HDAC4 (H-i+Den) and the denervation group interfering with HDAC4 and overexpressing MYOG (H-i/G-oe+Den). By detecting the wet weight ratio and muscle fiber cross-sectional area of mice in each group, it was found that the wet weight ratio and muscle fiber cross-sectional area of mice in the G-i+Den group were significantly higher than those in the denervated group (Figures 5A,B), which suggest that MYOG interference can also alleviate denervation-induced muscle atrophy. In the H-i/G-oe+Den group, the muscle wet weight ratio and muscle fiber cross-sectional area of mice were significantly lower than those of the H-i+Den group (Figures 5A,B), suggesting that overexpression of MYOG antagonized the effect of HDAC4 inhibition on denervation-induced muscle atrophy.

Western Blot results showed that MyHC expression changes were consistent with the trend of muscle wet weight ratio and muscle fiber cross-sectional area (Figure 5C). HDAC4 inhibition significantly reduced MYOG expression, suggesting that HDAC4 had a regulatory effect on MYOG expression. HDAC4 expression was significantly decreased after MYOG inhibition (Figure 5C), suggesting that MYOG may also regulate HDAC4 expression. To further investigate the role of HDAC4/MYOG in denervation-induced muscle atrophy, we examined the activity of the ubiquitin-proteasome system. The results showed that the expression levels of MuRF1 and MAFbx in skeletal muscle of mice in the G-i+Den group were significantly lower than those in the Dengroup (Figure 5D). However, the expression levels of MuRF1 and MAFbx in skeletal muscle of mice in the H-i/G-oe+Den group were significantly higher than those in the H-i+Den group (Figure 5D). TEM analysis showed that MOYG interference could also alleviate mitochondrial abnormalities caused by denervation. Compared with the H-i+Den group, mitochondrial vacuolar degeneration was more obvious in the H-i/G-oe+Den group (Figure 5E). These results indicate that MYOG inhibition can reduce the activity of the ubiquitin proteasome system, protect the functional and structural integrity of mitochondria, and effectively diminish denervation-induced muscle atrophy. Nevertheless, MYOG overexpression could decrease the protective effect of HDAC4 inhibition on denervation-induced muscle atrophy.

## Discussion

Peripheral nerve injury causes progressive atrophy of muscle fibers and a rapid decline in their function. In this study, we found a significant increase in HDAC4 expression in skeletal muscle after denervation. HDAC4 shRNA inhibited the expression of HDAC4 in denervated skeletal muscle, and it was found that down-regulation of HDAC4 could inhibit denervation-induced proteolysis, mitochondrial autophagy, and muscle fiber type transformation, thereby alleviating denervation-induced muscle atrophy.

HDAC4 is a member of an important epigenetic modifying enzyme family, which is involved in the formation and function of the central nervous system, bone and muscle (Sild and Booij, 2019). Muscle-specific HDAC4 knockdown impaired activation of the ubiquitin-proteasome system, decreased autophagy and inhibited oxidative stress in skeletal muscle (Pigna et al., 2018). Consistent with previous studies, our study also found that down-regulation of HDAC4 can inhibit denervation induced proteolysis, shift from slow-twitch to fast-switch, and mitophagy, accompanied by the decreased muscle specific E3 ubiquitin ligase (MuRF-1 and MAFbx) and autophagy-related proteins (ATG7, LC3B, PINK1 and BNIP3). Recent studies have shown that HDAC4 induces the deacetylation of Myosin heavy chain (MyHC), PGC-1α and heat shock cognate 71 kDa protein (HSC70). The selective inhibitor HDAC4 can reduce the degradation of MyHC and increase the protein level of PGC-1α in the process of muscle atrophy (Luo et al., 2019). Our study also found that HDAC4 knockdown reversed the decrease of MyHC, SIRT1, and PGC-1α induced by denervation and decreased muscle fiber type transformation. SIRT1 and PGC-1α could inhibit the activation of forkhead box protein O (FOXO) and nuclear factor-κB (NF-κB), and then restrain atrogene transcription (Cohen S. et al., 2015). Chalkiadaki et al demonstrated that SIRT1 could increase the levels of PGC-1α, markers of oxidative metabolism and mitochondrial biogenesis, decrease expression of the atrogenes, and exhibits a fiber shift from fast-to-slow twitch in a genetic model of Duchenne muscular dystrophy (Chalkiadaki et al., 2014). These results suggest that HDAC4 is not only involved in the process of muscular atrophy, but also plays an important role in maintaining specific metabolic processes of muscle fibers.

Transcriptome sequencing analysis suggests that HDAC4 may play a role by regulating MYOG, CDKN1A and SIK1. MYOG is a specific transcription factor in muscle, which plays an important role in the normal growth and development of muscle tissue. MYOG plays a key regulatory role in terminal myocyte differentiation (Mastroyiannopoulos et al., 2012). Loss of MYOG during the embryonic period can block embryonic development and lead to death (Berti et al., 2015). MYOG knockout also resulted in decreased muscle mass and muscle fiber cross-sectional area in mature individuals (Ganassi et al., 2018). However, in the muscle of adult mice, MYOG expression was maintained at a low level, and its activity was significantly enhanced after muscle denervation, suggesting that MYOG has other functions besides initiating myoblast differentiation. As we all know, the direct cause of muscle atrophy is that the balance between proteolysis and synthesis in muscle is broken, and the structural proteins in muscle tissue are hydrolyzed in large quantities and cannot be replenished, resulting in the decrease of muscle fiber diameter and loss of muscle mass (Huang et al., 2020; Wan et al., 2020). Our results indicate that MYOG is significantly overexpressed in denervated skeletal muscle, which may be related to its involvement in the activation of muscle-specific E3 ubiquitin ligase, which causes proteolysis and promotes muscular atrophy. Notably, overexpression of MYOG in the muscle of normal mice was not sufficient to cause muscle atrophy (data not shown), suggesting that the role of MYOG in skeletal muscle may be related to the microenvironment of skeletal muscle cells. Studies have shown that MYOG expression is only upregulated in neurogenic muscular atrophy, but not significantly increased in muscular atrophy caused by fasting or cancer cachexia (Moresi et al., 2010). Since E3 ubiquitin ligase (MuRF1 and MAFbx) are generally upregulated in a variety of skeletal muscle atrophy, the expression of MuRF1 and MAFbx must also be regulated by other molecules in fasting or cancer cachexia induced muscular atrophy. Our study also found that MYOG inhibition can reduce the activity of ubiquitin-proteasome hydrolysis pathway, protect the functional and structural integrity of mitochondria, and effectively diminish denervation-induced muscle atrophy, while overexpression of MYOG can weaken the protective effect of HDAC4 inhibition on denervation-induced muscle atrophy. These results suggest that HDAC4 is involved in denervation-induced muscle atrophy by regulating MYOG. These data were consistent with Mielcarek’s report that tHDAC4-myogenin axis was an important marker of Huntington’s disease (HD)-related skeletal muscle atrophy (Mielcarek et al., 2015). Dach2 is an inhibitor of MYOG. Knocking down Dach2 in innerved muscles causes an increase in MYOG, while overexpression of Dach2 can reduce denervation-induced injury (Méjat et al., 2005; Tang and Goldman, 2006). MacPherson et al found that HDAC4 is a negative regulator of Dach2, and the inactivation of HDAC4 will cause the increase of Dach2 (Tang et al., 2009). In conclusion, Dach2 can be used as a mediator of HDAC4-MYOG to participate in denervation-induced muscle atrophy, and the specific mechanism needs further experimental verification. Our data also showed that HDAC4 expression was significantly decreased after MYOG inhibition, suggesting that MYOG may also regulate the expression of HDAC4. However, this specific regulatory mechanism still needs to be further explored.

The epigenetic modification of histone deacetylases (HDACs) plays an important role in the regulation of gene transcription and plays an extremely important role in the life activities of cells. Transcriptome sequencing analysis suggests HDAC4 inhibition also causes changes in many genes related to cell cycle and cell division. Our study found that CDKN1A expression was up-regulated in denervated skeletal muscle and down regulated in HDAC4 inhibited skeletal muscle. CDKN1A is a cell cycle regulatory protein downstream of p53 gene, and it is involved in the regulation of cell growth, differentiation, aging and death (Xiao et al., 2020). Studies have shown that the expression of CDKN1A in the muscle of children with Duchenne muscular dystrophy is increased, and it is also up-regulated in the muscle of the elderly (Welle et al., 2004). In the starvation induced atrophy model, ATF4 can increase the expression levels of downstream CDKN1A and GADD45 α (Ebert et al., 2010). The expression of CDKN1A increased in the model of muscle atrophy induced by hind limb suspension, but decreased after reloading (Wang et al., 2017). Recent literature has reported that CDKN1A promotes muscle atrophy by reducing the expression of spermine oxidase (Adams et al., 2017). In conclusion, HDAC4/CDKN1A may be involved in the process of denervation-induced muscle atrophy by regulating the growth and differentiation of muscle cells. Growth arrest and DNA damage-inducible α (Gadd45α) participate in a variety of cellular processes, including maintenance of genomic integrity, growth arrest, apoptosis, senescence, and signal transduction. In skeletal muscle, GADD45α plays an important regulatory role in mitochondrial biogenesis and muscle atrophy (You et al., 2019). Sequencing data analysis also showed that the expression of GADD45α was significantly increased in skeletal muscle after denervation (FC = 44.903, *P* < 0.001). Interfering HDAC4 expression significantly decreased GADD45α expression in denervated target muscle (FC = 0.501, *P* < 0.001). Yoshihara et al demonstrated that induced the HDAC4/Gadd45α pathway was activated in atrophied muscles induced by hindlimb unloading. Exercise prior to hindlimb unloading may alleviate disuse muscle atrophy *via* inhibiting HDAC4/Gadd45α axis (Yoshihara et al., 2019). Bongers et al suggested that HDAC4 was an important regulator of Gadd45α in denervation-induced muscle atrophy (Bongers et al., 2013), which was consistent with our results. These studies indicate that HDAC4/Gadd45α axis is up-regulated in a variety of muscle atrophy models, including denervation induced atrophy, and plays an important regulatory role. This data provides new insights into the roles of the HDAC4/Gadd45α axis in skeletal muscle atrophy.

SIK1 acts as a key modulator in many physiological processes, including gluconeogenesis, cellular metabolism and growth (Hsu et al., 2020; Liu et al., 2020). Nixon et al suggested that SIK1 promotes insulin resistance on a high fat diet in skeletal muscle (Nixon et al., 2016). Insulin resistance plays an important role in many processes of muscular atrophy (Brener et al., 2020; Markov et al., 2020; Pár et al., 2021). Our study found that SIK1 expression was upregulated during denervation-induced muscle atrophy. Therefore, whether up-regulated SIK1 expression in denervated skeletal muscle is involved in muscular atrophy by promoting insulin resistance needs to be further explored. Stewart et al demonstrated that during differentiation of primary myogenic progenitor cells, SIK1 was significantly induced for the activation of MEF2. Depletion of SIK1 remarkably impairs myogenic differentiation (Stewart et al., 2013). SIK1, a class IIa HDAC kinase, participates in the regulation of a variety of HDACs. The function of HDAC4 is controlled by the activity of SIK1 (Stewart et al., 2013). In our study, it was found that SIK1 expression was up-regulated during denervation-induced muscle atrophy and down-regulated in skeletal muscle after HDAC4 inhibition. Thus it can be seen that SIK1 may also be regulated by HDAC4, that is, there is a mutual regulation mechanism between HDAC4 and SIK1, but the exact molecular mechanism still needs to be further explored. In conclusion, HDAC4/SIK1 axis may also play an important regulatory role in denervation-induced muscle atrophy.

In conclusion, HDAC4 plays an important role in denervation-induced muscle atrophy. HDAC4 inhibition alleviates denervation-induced muscle atrophy partially through blocking myogenin-dependent atrogenes activation. HDAC4 also regulates cell division, cell cycle, apoptotic process, and cell differentiation *via* CDKN1A and SIK1, involving in denervation-induced muscle atrophy. This study not only enriched the molecular mechanism of denervation-induced muscle atrophy, but also provided an important target for the prevention and treatment of denervation-induced muscle atrophy and innovative drug development.

## Data Availability Statement

The datasets presented in this study can be found in online repositories. The names of the repository/repositories and accession number(s) can be found in the article/Supplementary Material.

## Ethics Statement

The animal study was reviewed and approved by the Animal Care Guidelines of Nantong University and approved by Jiangsu Provincial Laboratory Animal Management Committee.

## Author Contributions

HS: conception and design. HS, XY, and JZ: administrative support. WM, YC, XY, YS, XC, LZ, YJ, ZC, and JZ: provision of study materials or patients. WM, YC, XY, YS, XC, LZ, YJ, ZC, JZ, and HS: collection and assembly of data. WM, XY, YS, YC, XC, and HS: data analysis and interpretation. All authors contributed to the article and approved the submitted version.

## Conflict of Interest

The authors declare that the research was conducted in the absence of any commercial or financial relationships that could be construed as a potential conflict of interest.

## Supplementary Material

SUPPLEMENTARY FIGURE 1 Effect of HDAC4 interference on MYOG expression. Mice in sham operation group (Sham) were injected with empty vector virus into tibialis anterior muscle. The sciatic nerve transection model was prepared after injection of HDAC4-shRNA lentivirus (H-i+Den), empty vector virus (Den) into the tibialis anterior muscle of mice 3 days. Protein levels of MYOG in muscle were detected by Western blot after denervation for 14 days. ****P* < 0.001 vs. sham group. ^##^*P* < 0.01 vs. denervated group.

SUPPLEMENTARY FIGURE 2 Overexpression of MYOG causes atrophy of the C2C12 myotubes. C2C12 was transfected with empty vector lentivirus (Ctrl) and MYOG overexpression lentivirus (G-OE) after induced differentiation for 3 days, respectively. After 3–4 days, **(A,B)** MHC staining was used to analyze the diameter of the C2C12 myotubes. **(C)** The expression levels of MYOG, MyHC, MuRF1 and MAFbx were detected by Western Blot. **P* < 0.05 vs. Ctrl group.
