# Supplementary figures and images for: HDAC4 Knockdown Alleviates Denervation-Induced Muscle Atrophy by Inhibiting Myogenin-Dependent Atrogene Activation
Source: Front Cell Neurosci. 2021 Jun 30;15:663384. doi: 10.3389/fncel.2021.663384 (PMC8278478; doi:10.3389/fncel.2021.663384)

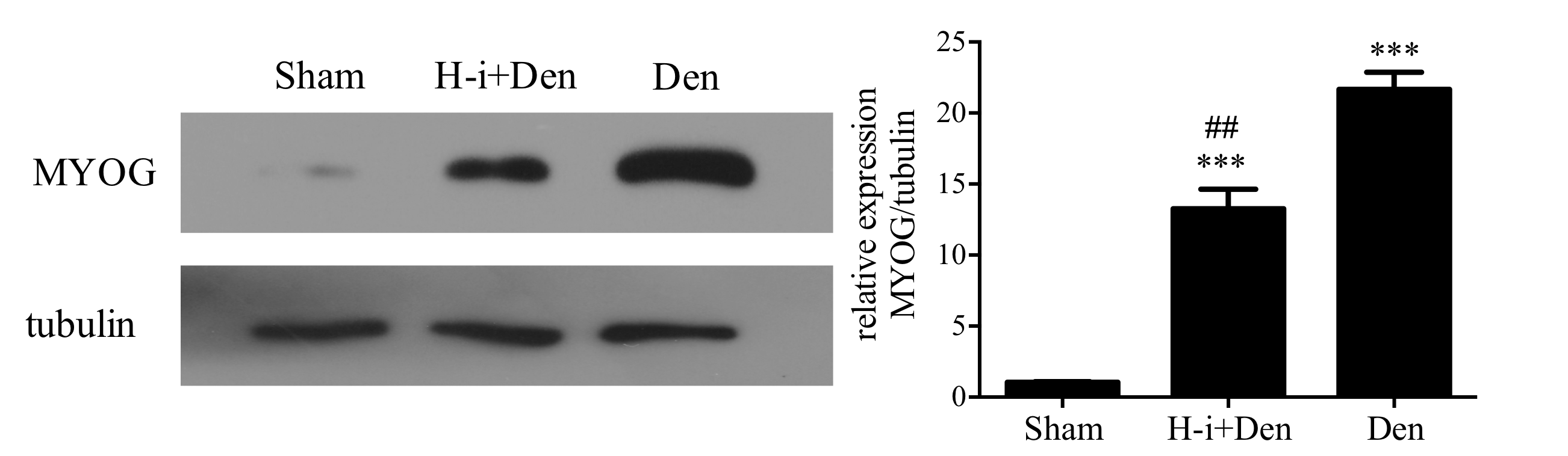

Supplement: SUPPLEMENTARY FIGURE 1 — Effect of HDAC4 interference on MYOG expression. Mice in sham operation group (Sham) were injected with empty vector virus into tibialis anterior muscle. The sciatic nerve transection model was prepared after injection of HDAC4-shRNA lentivirus (H-i+Den), empty vector virus (Den) into the tibialis anterior muscle of mice 3 days. Protein levels of MYOG in muscle were detected by Western blot after denervation for 14 days. ***P < 0.001 vs. sham group. ##P < 0.01 vs. denervated group. [file Image_1.TIFF]

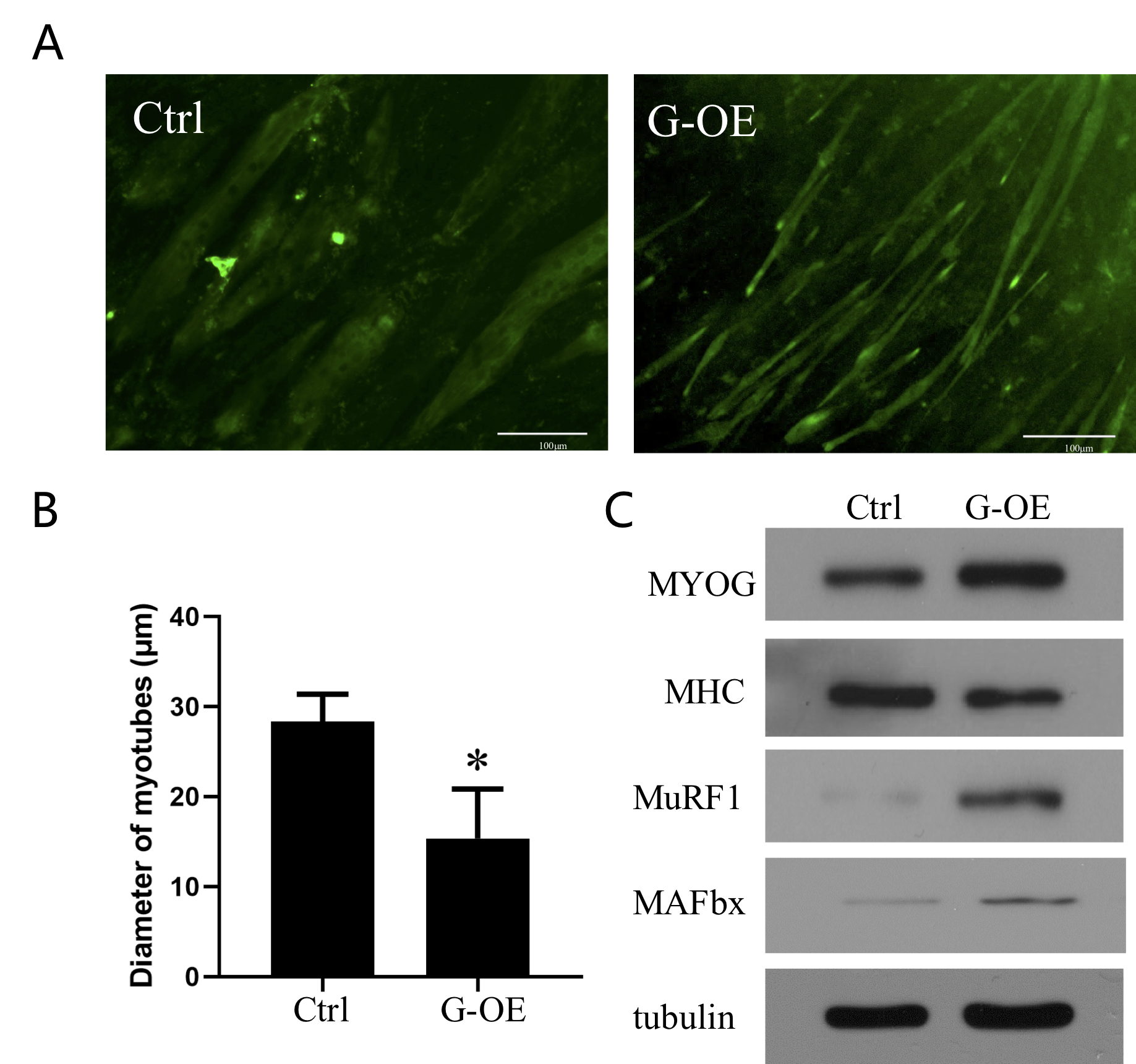

Supplement: SUPPLEMENTARY FIGURE 2 — Overexpression of MYOG causes atrophy of the C2C12 myotubes. C2C12 was transfected with empty vector lentivirus (Ctrl) and MYOG overexpression lentivirus (G-OE) after induced differentiation for 3 days, respectively. After 3–4 days, (A,B) MHC staining was used to analyze the diameter of the C2C12 myotubes. (C) The expression levels of MYOG, MyHC, MuRF1 and MAFbx were detected by Western Blot. *P < 0.05 vs. Ctrl group. [file Image_2.tiff]
